# Supplementary material for: Unimodal head-width distribution of the European eel (Anguilla anguilla L.) from the Zeeschelde does not support disruptive selection
Source: PeerJ. 2018 Nov 6;6:e5773. doi: 10.7717/peerj.5773 (PMC6225841; doi:10.7717/peerj.5773)
Supplement: Supplemental Information 1 [file peerj-06-5773-s001.docx]

Unimodal head-width distribution of the European eel (*Anguilla anguilla* L.) from the Zeeschelde does not support disruptive selection

Pieterjan Verhelst^1,2,3,4,*^, Jens De Meyer^5^, Jan Reubens^3^, Johan Coeck^4^, Peter Goethals^2^, Tom Moens^1^, Ans Mouton^4^

^1^ Marine Biology Research Group, Ghent University, Krijgslaan 281, 9000 Ghent, Belgium

^2^ Laboratory of Environmental Toxicology and Aquatic Ecology, Ghent University, Coupure Links 653, 9000 Ghent, Belgium

^3^ Flanders Marine Institute, Wandelaarkaai 7, 8400 Ostend, Belgium

^4^ Research Institute for Nature and Forest (INBO), Havenlaan 88, bus 73, 1000 Brussels, Belgium

^5^ Evolutionary Morphology of Vertebrates, University Ghent, K.L. Ledeganckstraat 35, 9000 Ghent Belgium

* Corresponding author

Pieterjan Verhelst

Ghent University - Biology Department

Marine Biology Research Group

Krijgslaan 281 / S8

9000 Gent - Belgium

Tel.: +32 (0)9 264 85 17

[Pieterjan.Verhelst@UGent.be](mailto:Pieterjan.Verhelst@UGent.be)

Appendix

In this appendix, we give a detailed description of the applied methodology to determine the head width distribution of the eels based on the unstandardized residuals.

First, linear regressions of head width (**Fig. 1**) and head length to total length (**Fig. 2**) revealed a significant difference between the two slopes (i.e. under the null hypothesis that slopes are equal, *p* < 0.05). Despite the fact that the regression of head length had a steeper slope than the regression of head width, head width / head length increased with total length (**Fig. 3**). Next, head width / total length (**Fig. 4**) and head length / total length were plotted over total length (**Fig. 5**) and revealed that the latter slightly decreased, while head width / total length increased. Further, variation of the former proved larger than variation of the latter (range: 0.03 - 0.06 and 0.09-0.19 respectively). Since head length / total length only slightly increased with total length and the variation was lower compared to head width / head length, we can assume that head length increases proportionally with total length. However, for head width / total length this is not the case due to plasticity in head morphology and a size effect. Consequently, to correct for these issues, the unstandardized residuals of head width / head length over total length were calculated and used for analysis of the eels’ head width distribution.


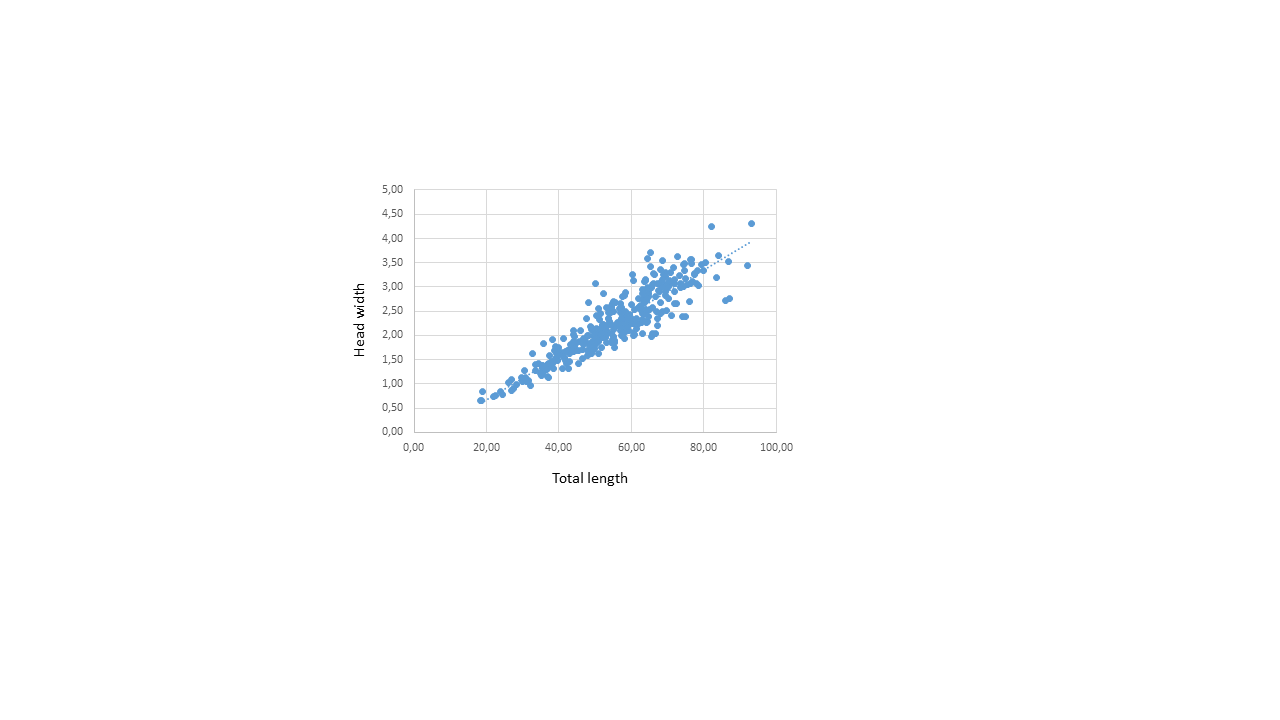


**Figure 1.** Head width over total length (y = 0.0447x – 0.2104, R² = 0.859)


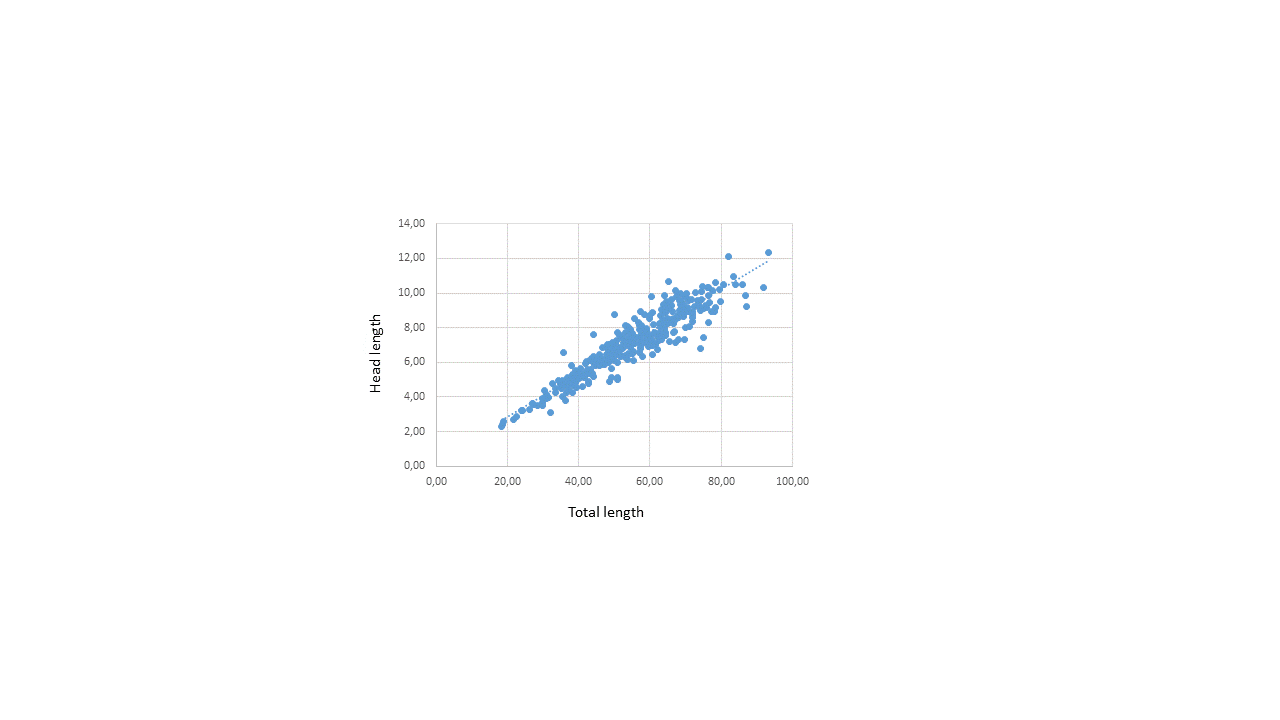


**Figure 2.** Head length over total length (y = 0.1227x + 0.3906, R² = 0.818).


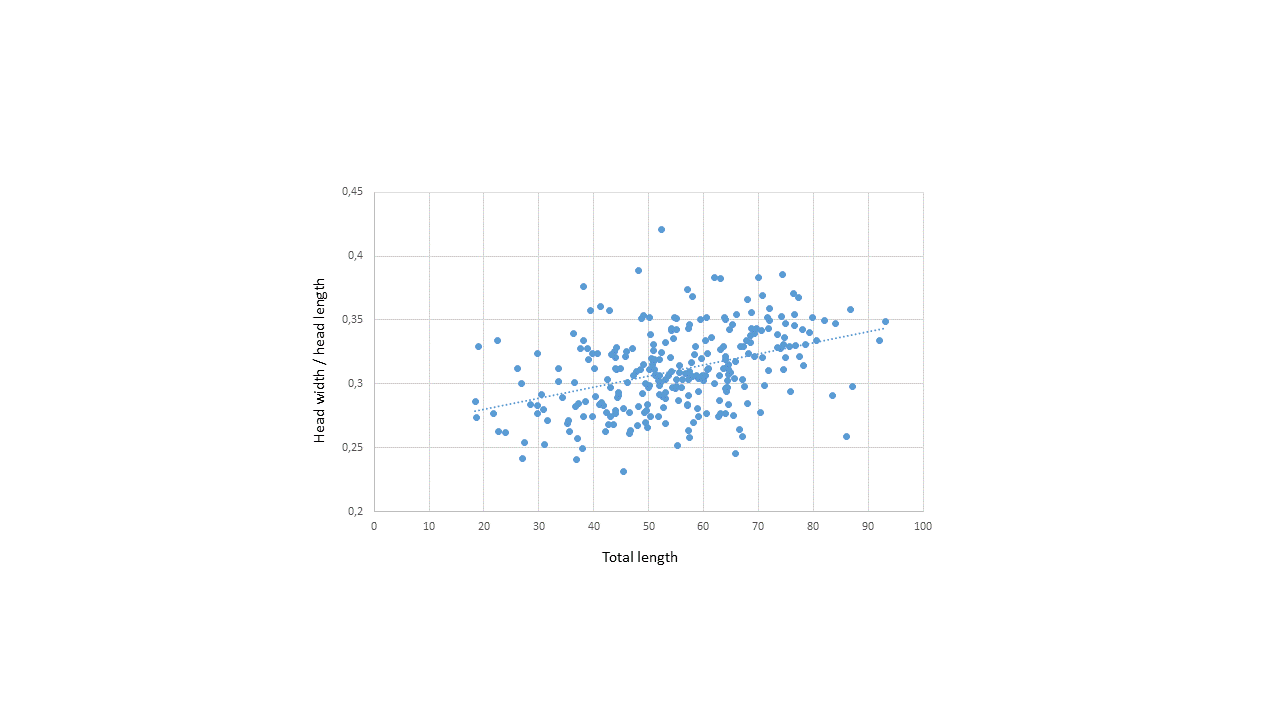


**Figure 3.** Head width / head length over total length (y = 0.0010x + 0.2592, R² = 0.160).


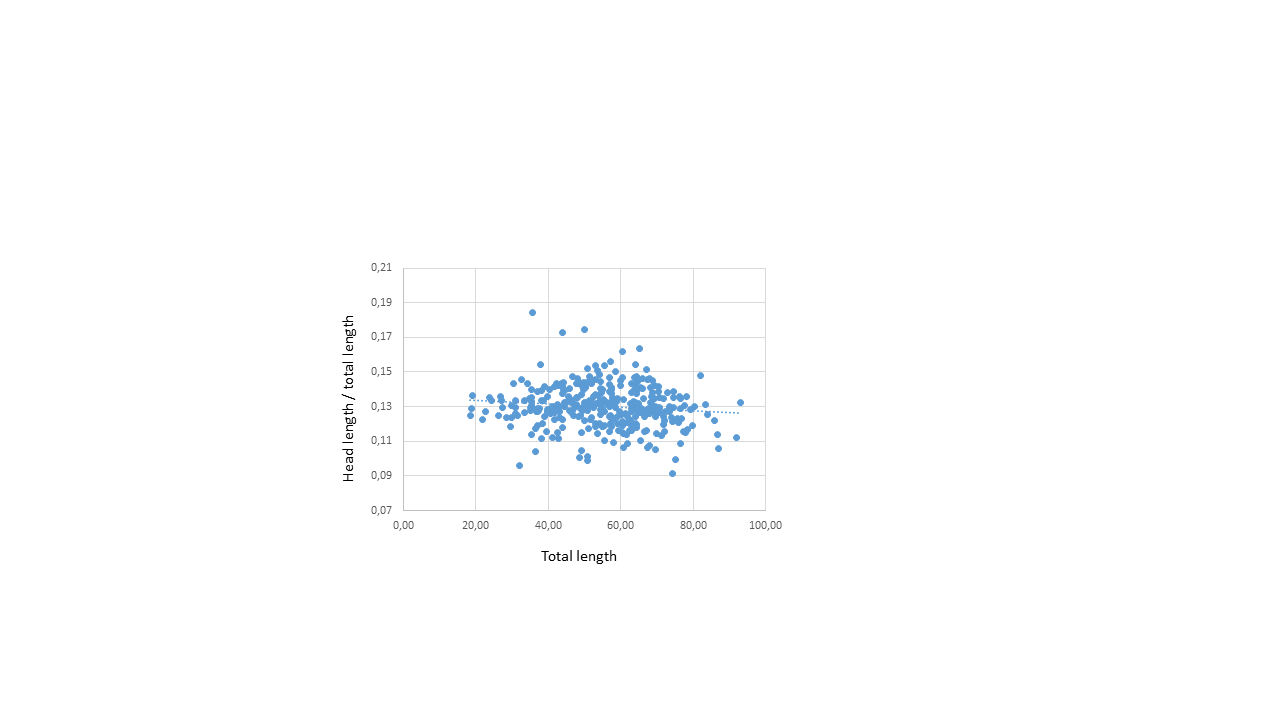


**Figure 4.** Head length / total length over total length (y = 1e^-4^x + 0.0353, R² = 0.092).


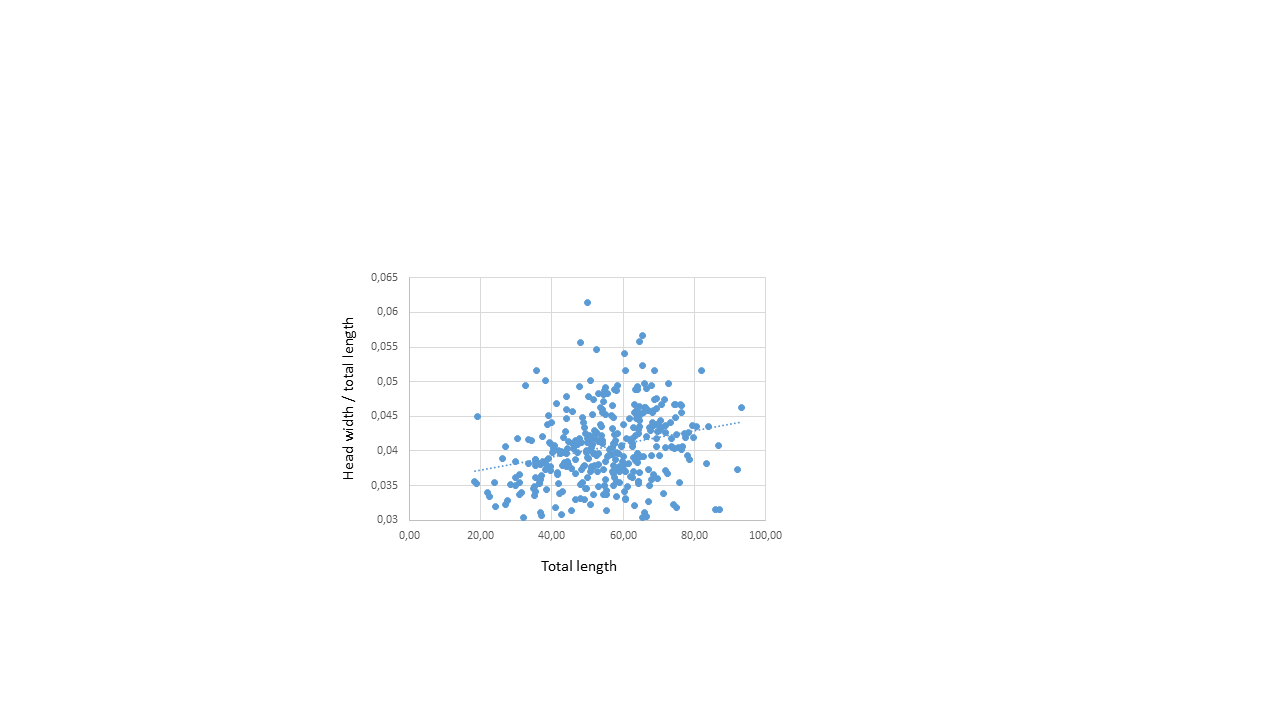


**Figure 5.** Head width / total length over total length (y = -1e^-4^x + 0.1354, R² = 0.027).
