# Supplementary material for: Unimodal head-width distribution of the European eel (Anguilla anguilla L.) from the Zeeschelde does not support disruptive selection
Source: PeerJ. 2018 Nov 6;6:e5773. doi: 10.7717/peerj.5773 (PMC6225841; doi:10.7717/peerj.5773)
Supplement: Table S1 [file peerj-06-5773-s003.docx]

**Table S1.** Values of the model selection criteria AIC and BIC for a unimodal and bimodal distribution for 50%, 75% and 90% of the data.

| Percentage of the dataset (%) | Model selection criterion | Unimodal | Bimodal |
| --- | --- | --- | --- |
| 50 | AIC | -604 | -600 |
|  | BIC | 598 | 588 |
| 75 | AIC | -858 | -857 |
|  | BIC | 851 | 843 |
| 90 | AIC | -1032 | -1034 |
|  | BIC | 1025 | 1020 |
